# Predictive value of proteomic markers for advanced rectal cancer with neoadjuvant chemoradiotherapy

**DOI:** 10.1186/s12885-022-09960-z

**Published:** 2022-08-09

**Authors:** Hanyang Wang, Dengbo Ji, Huifang Tian, Zhaoya Gao, Can Song, Jinying Jia, Xinxin Cui, Lijun Zhong, Jing Shen, Jin Gu

**Affiliations:** 1 Key Laboratory of Carcinogenesis and Translational Research, (Ministry of Education), Department of Gastrointestinal Surgery III, Peking University Cancer Hospital & Institute, No. 52 Fucheng Rd, Haidian District, Beijing, 100142 China; 2 Central Laboratory, Peking University Cancer Hospital & Institute, Beijing, China; 3 Peking University S.G. Hospital, Beijing, China; 4 School of Life Sciences, Tsinghua University, Beijing, 100084 China; 5 Peking-Tsinghua Center for Life Sciences, Beijing, China; 6 Medical and Health Analytical Center, Peking University Health Science Center, Beijing, 100191 China

**Keywords:** Rectal cancer, Neoadjuvant chemo/radiotherapy, Isobaric tags for relative and absolute quantitation, Parallel reaction monitoring, Prognosis

## Abstract

**Background:**

Preoperative neoadjuvant chemoradiation (nCRT) has been the standard treatment for locally advanced rectal cancer. Serum biomarkers to stratify patients with respect to prognosis and response to nCRT are needed due to the diverse response to the therapy.

**Methods:**

Thirteen paired pre- and post-nCRT sera from rectal cancer patients were analyzed by isobaric tags for relative and absolute quantitation (iTRAQ) method. Twenty-five proteins were selected for validation by parallel reaction monitoring (PRM) in ninety-one patients.

**Results:**

Totally, 310 proteins were identified and quantified in sera samples. Reactome pathway analysis showed that the immune activation-related pathways were enriched in response to nCRT. Twenty-five proteins were selected for further validation. PRM result showed that the level of PZP was higher in pathological complete response (pCR) patients than non-pCR patients. The Random Forest algorithm identified a prediction model composed of 10 protein markers, which allowed discrimination between pCR patients and non-pCR patients (area under the curve (AUC) = 0.886 on testing set). Higher HEP2 and GELS or lower S10A8 in baseline sera were associated with better prognosis. Higher APOA1 in post nCRT sera was associated with better disease-free survival (DFS).

**Conclusions:**

We identified and confirmed a 10-protein panel for nCRT response prediction and four potential biomarkers HEP2, GELS, S10A8 and APOA1 for prognosis of rectal cancer based on iTRAQ-based comparative proteomics screening and PRM-based targeted proteomic validation.

**Supplementary Information:**

The online version contains supplementary material available at 10.1186/s12885-022-09960-z.

## Background

nCRT followed by surgery with a total mesorectal excision is the recommended treatment for patients with locally advanced rectal cancer. nCRT has been proven to be effective to downstage the primary tumor which may facilitate complete resection with negative surgical margins and significantly reduce local recurrence [1, 2]. The efficacy of nCRT varies among individual patients ranging from pathological complete response (pCR; defined as ypT0N0) to progression of disease. About 10–25% of patients treated with nCRT achieve a pCR [3], which is associated with low rates of local and distant recurrence and better overall and disease-free survival compared with patients without pCR [4, 5]. However, distant recurrence rate of all patients treated with curative regime has not decreased significantly even though local recurrence and overall survival (OS) have improved. It is estimated that around 30% of patients will eventually develop distant metastases [6]. To find useful biomarkers to stratify patients with respect to prognosis and response to nCRT is clinically important. The nCRT response is not predictable by using the pathological and radiological features of the primary cancer [7]. Many studies have been devoted to identify predictive factors present in tumors before treatment. However, gene expression studies have failed to provide reproducible and clinically useful information for the identification of patients who could benefit from nCRT [8].

Circulatory predictive biomarkers would be ideal because of the feasibility with minimum invasion. Proteomic analysis by mass spectrometry (MS) is largely used to explore the proteins in blood samples which are associated with cancer incidence and progression. Among of the proteomic technologies, iTRAQ-based proteomic strategies have the advantage of high throughput over other methods and can provide quantification information and identification of peptides at the same time [9]. Targeted proteomic techniques which involve LC–MS/MS analyses in multiple-reaction monitoring (MRM) or parallel-reaction monitoring (PRM) mode enable high-throughput quantification of biomarkers and are predicted to replace antibody-based assays [10]. PRM provides better accuracy and specificity for quantifying analysis in complex sample matrices, which can avoid the defects of antibody-dependent analyses, such as variable protein glycosylation as observed in cancer [11].

In this study, we used the iTRAQ method to identify sera proteins associated with response to nCRT in rectal cancer patients. To validate these proteins as specific biomarkers, we used a PRM-based targeted proteomic technique to determine their levels and evaluate their prognostic value.

## Methods

### Patients

This study was approved by the Ethics Review Committees of Peking University Cancer Hospital & Institute and in accordance with the Declaration of Helsinki. All patients were informed prior to the study and a consent form was signed by each participant. Written informed consent was obtained from all patients. There were two patient cohorts. In the first, biomarker candidates were identified in the explorative cohort (*n* = 13). This retrospective cohort consisted of well-characterized response and non-response to nCRT rectal cancer patients. Biomarkers were then tested in a training cohort (*n* = 91). All patients underwent surgical resection from 2014 to 2016.

Inclusion criteria were: 1. diagnosis of rectal adenocarcinoma by biopsy; 2. tumor staged as T3-4 or any T, N + by endorectal ultrasonography, pelvic magnetic resonance imaging, or computed tomography (CT) and 3. no evidence of distant metastasis.

### Treatment

All patients were treated with long-course nCRT followed by TME surgery. Radiotherapy was delivered as fixed field intensity-modulated radiation therapy (IMRT) 50.6 Gy to the tumor bed and 41.8 Gy in 22 fractions to the clinical target volume (CTV). The CTV refers to the primary tumor regional lymph nodes and extending to the promontory. Capecitabine treatment was administered concurrently with IMRT at a dose of 825 mg/m^2^ orally twice per day. TME surgery was recommended 8–10 weeks after nCRT. Adjuvant chemotherapy was recommended to the patients. Capecitabine alone, mFOLFOX6 or CapeOx were prescribed at the discretion of the physician. CEA serum levels and CT scans of the chest, abdomen and pelvis were performed at baseline, 8 weeks after nCRT and then every 6 to 12 months after treatment. A summary of the clinical characteristics of these patients is shown in Additional file 1, Supplementary Table 1, 2.

### Assessment of treatment response and tumor downstaging

The 8th edition of the American Joint Committee on Cancer TNM system was used for pathological staging [12]. Neoadjuvant radiotherapy effect was evaluated after surgery by specialized gastrointestinal pathologists using TRG system as follows: Grade 0: complete regression, no tumor cells; Grade 1: single or small groups of tumor cells, moderate response; Grade 2: residual cancer outgrown by fibrosis, minimal response; Grade 3: minimal or no tumor cells killed, poor response.

### Blood samples

Blood samples were collected at baseline and after nCRT. Serum was span at 3000 × g for 5 min at 4 °C to remove debris. Samples were aliquoted and stored at − 80 °C and processed within 1 h based on protocols of NCI's Early Detection Research Networks (EDRN) [13]. All samples were checked for hemolysis using the Harboe's spectrophotometric methods [14] and hemolytic samples with free hemoglobin concentration > 0.6 g/L [15, 16] were excluded.

### Protein depletion, digestion and iTRAQ labeling

Abundant proteins were removed from sera using a Protein Extract Albumin IgG removal kit following the manufacturers' protocol (Merck Millipore, Darmstadt, Germany). Depleted samples were digested according to the manufacturer’s protocol for filter-aided sample preparation (FASP). In brief, 200 μg of each sample was mixed with 200 μL of 8 M urea in 0.1 M Tris/HCl (pH 8.5) in a Vivacon 500 filtrate tube (Cat No. VN01H02, Sartorius Stedim Lab Ltd, Stonehouse, GL10 3UT, UK) and was centrifuged at 13 000 × g at 20 °C for 15 min. The sample was washed twice by adding 200 μL of 8 M urea in 0.1 M Tris/HCl (pH 8.5) and then centrifuged to remove irrelevant substances. Afterward, 10 μL of 0.05 M Tris-(2-carboxyethyl) phosphine (TCEP) in water was added to the filters, the sample was incubated at 37 °C for 1 h. Then, 10 μL of 0.1 M iodoacetamide (IAA) was added to the filter, and the sample was incubated in darkness for 30 min. The filter was washed twice with 200 μL of 50 mM NH_4_HCO_3_. Finally, 5 μg of trypsin (Promega, Madison, WI, USA) in 100 μL of 50 mM NH_4_HCO_3_ was added to each filter. Samples were digested with trypsin (1:40 [w/w] (sequence grade modified; Promega, Madison, WI, USA) overnight at 37 °C. Peptides were collected by filtration. Peptide content was estimated by nanodrop-microvolume-spectrophotometer [17, 18].

The iTRAQ 8-plex reagents (AB Sciex Pte. Ltd., Framingham, MA, USA) were used to label the samples as follows: pre-nCRT sera for resistant group (iTRAQ reagent 113); post nCRT for resistant group (iTRAQ reagent 114); pre-nCRT for response group (iTRAQ reagent 115); and post nCRT for response group (iTRAQ reagent 116). The four sample groups were incubated for 2 h at room temperature. The resulting labeled peptide samples were then pooled before chromatographic fractionation.

### High pH reverse phase chromatography

The labeled samples were resuspended in buffer A (2% acetonitrile, 98% water with ammonia at pH 10) and fractionated by Agilent 1100 HPLC system (Agilent Technologies, Waldbronn, Germany). The samples were loaded onto a 250 mm × 4.6 mm 5 μm C_18_ HPLC columns (Bonna-Agela, Catalog Number: DC952505-0, Tianjin, China). Peptides were eluted by the application of a linear 45 min gradient up to 95% buffer B (2% water, 98% acetonitrile with ammonia at pH 10) with 40 × 60 s fractions collected from 5 min. Fractions containing eluted peptides were pooled into 15 fractions based on peptide density [19]. Finally 10 fractions were collected, and each fraction was dried in Eppendorf Concentrator plus (Eppendorf, Hamburg, Germany) at 45 °C and stored at − 80 °C.

### Mass spectrometry (MS/MS)

The peptides were redissolved with 0.1% formic acid (FA) and analyzed on a Q-Exactive HF mass spectrometer coupled with an Easy-nLC 1000 instrument (Thermo Fisher Scientific, Waltham, MA, USA). The mass spectrometer was operated in the data-dependent mode with positive polarity at electrospray voltage of 2 kV. Full scan MS spectra (m/z 400–1500) were acquired followed by MS/MS on the top 20 intense ions detected.

The mass spectrometry (MS) raw data were analyzed with Proteome Discoverer software (version 1.4) using the Mascot search engine to search against the human database (UniProt, release 2020_01) with the following settings: 1) one missed cleavage allowed, 2) precursor tolerance 7 ppm, fragment ion tolerance 0.5 Da, 3) carbamidomethylated cysteine as a fixed modification, oxidized methionine and acetyl (Protein N-term) as variable modification. Peptides with peptide score ≥ 10 and FDR < 0.01 (based on the target-decoy database algorithm) were used for protein grouping. Protein groups identified ≥ 2 peptides from all samples were considered for further analysis and only unique peptides were used for protein quantification (Supplementary Table 3).

Proteins with a fold change larger than 1.5 or less than 0.667 in at least one comparison were selected as significantly differential expressed proteins (Supplementary Table 4). The enriched pathway analysis was performed with Reactome pathway analysis (www.reactome.org).

Biomarker candidates for nCRT sensitivity and outcome were determined based on differences in protein identifications and relative protein abundances between the resistant and response groups at baseline, post treatment or between pre and post nCRT groups. From the selected biomarkers, peptides suitable for quantification were identified.

### Quantification of targeted biomarkers using parallel reaction monitoring

On the basis of the results of the explorative analysis, a targeted, quantitative MS analysis was performed using parallel reaction monitoring. In this method, the mass spectrometer is programmed to select only the mass/charge windows corresponding to the peptides of interest. All their fragment ions are then scanned.

Protein samples were digested according to the manufacturer’s protocol for FASP. Protein samples (200 μg) from each serum were processed according to the manufacturer’s protocol for FASP. PRM analyses were performed on a Quadrupole-Orbitrap LC–MS instrument platform (Thermo Fisher Scientific, Waltham, MA, USA). The PRM scan mode consisted of one full scan (resolution of 15,000 at m/z = 200, AGC target = 3e6, maximum injection time = 10 ms, scan range = 300-1800 m/z) and sequential PRM scans (resolution of 15,000 at m/z = 200, AGC target = 5e5, maximum injection time = 50 ms, isolation window = 2 m/z, NCE = 27). Three to five peptides of the proteins HPT, HPTR, CO5, APOA1, APOC1, APOC3, SAMP, APOH, A2GL, FINC, RET4, CXCL7, APOB, HRG, S10A8, HEP2, GELS, APOA4, APOA, CO4A, PZP, ZA2G, KAIN, PEDF and FHR1 were targeted (see Supplementary Table 5 for peptide sequence information). The retention times of the selected precursors were extracted from a data-dependent acquisition (DDA) data. The chromatographic separation was 5%-44% B (80% ACN) from 5 to 45 min using a flow rate of 300uL/min, and the retention time windows for the selected precursors were of 3 min. Extraction of fragment ion chromatograms was performed in Skyline (version 3.7.0.10940). The transition settings were as follows: ion charges: 1,2; ion types, y, b, p; product ion selection from m/z > precursor to 3 ions. Protein quantities were calculated using MSstats plugin in Skyline (version 3.5) as the integrated fragment ion peak areas (XIC) of three transitions of two to three peptides for the target proteins.

### TCGA data analysis

Kaplan − Meier survival analysis was performed using CRC RNA-seq data from the Cancer Genome Atlas presented in the Human Protein Atlas (www.proteinatlas.org), where patients were stratified based on the expression levels of GELS, HEP2, S10A8 genes being among the top (high group) and bottom (low group) quartiles, respectively. CRC tissues RNA-seq data from TCGA are derived from tumor tissue. Differences in survival with log rank p-values being less than 0.05 were considered significant.

### Statistical analysis

All data were presented as the mean ± SE and were analyzed using GraphPad Prism v.9. We used Benjamin-Hochberg’s false discovery rate method for a correction for multiple testing. All proteins (*n* = 25) from the PRM experiment were accounted for, when adjusting for multiple testing. q values less than 0.05 were considered significant. Differences in clinicopathological characteristics between high- and low- level sera biomarkers were assessed using Chi-square test. To evaluate the predictive potential of the selected proteins for nCRT response, ROC and AUC were calculated. DFS was measured from date of surgery to documented first recurrence or death as a result of colorectal cancer, and was censored at last follow-up or non-rectal cancer-related death. OS was defined as the time from the date of surgery until death from any cause. The DFS and OS curves were plotted using Kaplan–Meier analysis, and the log–rank test was used to detect the significant difference between the groups. All tests were two-sided and the level of statistical significance was set at *p* < 0.05.

Combined prediction model was developed using the Random Forest (RF) algorithm. To select the best features from all 25 biomarkers, SelectKBest was used in Scikit-learn, removing less important features from the dataset. Chi2 method is used as the scoring function. We used the Random Forest algorithm to classify the data. 70% of the data was used as the training set and the rest as the testing set. In order to cross verify the prediction probability of feature combinations, ten-fold cross validation was used to obtain a robust classification model. All calculations were performed using Python version 3.7.6 and Sckit-Learn version 1.0.2

## Results

### Identification and quantification of proteins in sera with iTRAQ experiments

The iTRAQ peptide labeling efficiency was 98.61%. We identified and quantified 310 proteins from sera of 13 paired pre- and post-nCRT rectal cancer patients by iTRAQ (Supplementary Table 3). The samples were grouped and compared as follows: baseline resistant to nCRT group (*n* = 6); post treatment for resistant to nCRT group (*n* = 6); baseline for response to nCRT group (*n* = 7); and post treatment for response to nCRT group (*n* = 7) (labeled as 113, 114, 115 and 116 iTRAQ reagents, respectively). After data analysis with Proteome Discoverer software, we identified a total of 310 proteins. Proteins with more than 1.5-fold changes were considered to be significant (Supplementary Table 4). This analysis generated 9 upregulated and 18 down-regulated proteins in baseline resistant group compared with response group. In post nCRT sera, there were 17 up-regulated and 21 down-regulated proteins in the resistance group compared with the sensitive group. Significant changes were also observed between pre- and post-nCRT. For resistant group, there were 17 up-regulated and 27 down-regulated proteins in the post-nCRT sera as compared with the pre-nCRT samples. For response group, there were 7 up-regulated and 24 down-regulated proteins in the post-nCRT sera as compared with the pre-nCRT.

### Reactome enrichment analysis of sera proteome

To identify biological function associated with the response to nCRT, Reactome pathway analysis was performed with the differentially expressed proteins in baseline sera. Our results showed that the immune activation-related pathways were enriched, including classical antibody-mediated complement activation, FCGR activation, FCGR3A-mediated IL10 synthesis, Innate Immune System, FCGR dependent phagocytosis, Interleukin-4 and Interleukin-13 signaling, Immune System, CD22 mediated BCR regulation, Antigen activates B Cell Receptor (Table 1). We also analyzed the biological function associated with the response to nCRT in post-nCRT sera and found that the immune activation-related pathways were also enriched, especially the signaling by interleukins (Table 1).

**Table 1:** Reactome pathway analysis of baseline response vs. resistant to nCRT

Baseline response vs. resistant to nCRT			Post-nCRT response vs. resistant group		
Pathway name	Entities *p* Value	Entities FDR	Pathway name	Entities *p* Value	Entities FDR
Classical antibody-mediated complement	9.83E-08	9.93E-06	Interleukin-4 and Interleukin-13 signaling	6.96E-12	2.35E-09
FCGR activation	4.08E-06	8.98E-05	Immune System	4.87E-09	8.19E-07
FCGR3A-mediated IL10 synthesis	1.84E-05	2.95E-04	Binding and Uptake of Ligands by Scavenger Receptors	1.16E-07	7.77E-06
Innate Immune System	2.75E-05	3.48E-04	Degradation of the extracellular matrix	8.00E-07	3.36E-05
Binding and Uptake of Ligands by Scavenger Receptors	4.23E-05	4.65E-04	Innate Immune System	1.32E-06	4.89E-05
Fcgamma receptor (FCGR) dependent phagocytosis	8.12E-05	8.12E-04	Signaling by Interleukins	1.52E-06	5.01E-05
Interleukin-4 and Interleukin-13 signaling	0.001422	0.011379	Classical antibody-mediated complement activation	2.23E-05	5.58E-04
Immune System	0.001719	0.013749	Cytokine Signaling in Immune system	6.51E-05	0.001107
CD22 mediated BCR regulation	0.012231	0.071155	Regulation of Insulin-like Growth Factor (IGF) transport and uptake by Insulin-like Growth Factor Binding Proteins (IGFBPs)	7.94E-05	0.001271
Antigen activates B Cell Receptor (BCR) leading to generation of second messengers	0.023937	0.10811	FCGR activation	4.55E-04	0.004546

### Identification and validation of protein and peptide biomarkers by PRM

**Fig. 1:**
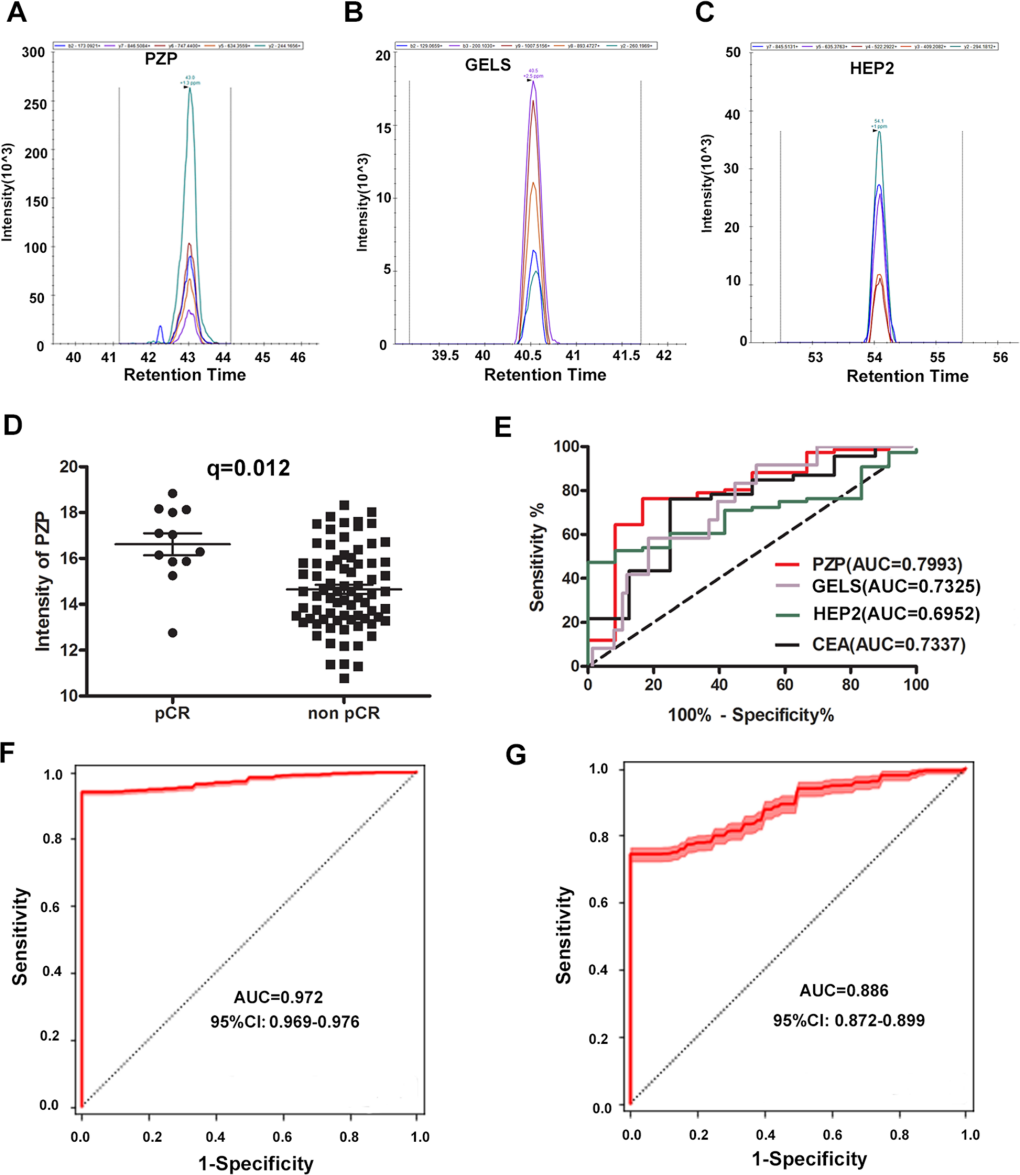
Sera levels of several proteins in response and resistant to nCRT groups of rectal cancer. **A**-**C** PRM traces for the quantification of PZP, GELS and HEP2. **D** Scatter plots of sera PZP concentration obtained from pCR and non-pCR patients using the PRM. **E** Receiver operating characteristic (ROC) curve for nCRT response by the baseline PZP, GELS, HEP2 and CEA. ROC curves generated using the response information and levels of the baseline PZP, GELS, HEP2 and CEA are able to discriminate between pCR patients and non-pCR. PZP has the strongest predictive value (area under the curve [AUC] = 0.7993) to discriminate those patients. **F** ROC analysis for the training set using Random Forest. **G** ROC analysis for the testing set using Random Forest

Biomarker candidates for the response to nCRT were determined based on the differences in protein identifications and relative protein abundances between the relevant samples, from which 25 proteins were selected. From the selected biomarkers, peptides suitable for quantification were identified (Supplementary Table 5) and validated by PRM. The PRM traces for the quantification of PZP, GELS and HEP2 are shown in Fig. 1A-C, respectively. In the validation cohort, the level of PZP was higher in pCR patients than in non-pCR patients (q = 0.012) (Fig. 1D).

There was no significant difference in GELS (q = 0.107) and HEP2 (q = 0.107) levels between the two groups (Supplementary Fig. 1A-B). The patients were divided into two groups by assessing tumor regression grade (TRG) in the resected specimens. TRG 0 and 1 were defined as responding group, TRG 2 and 3 were non-responding group. The levels of GELS and HEP2 in baseline sera also showed no significant difference between the responding group and the non-responding group (q = 0.246 and q = 0.427, respectively) (Supplementary Fig. 1C-D).

Using ROC curve analysis, the predictive performance of individual markers in distinguishing pCR patients from non-pCR was analyzed by the AUC values. ROC analyses revealed that the baseline PZP level was a useful predictive marker for differentiating pCR patients from non-pCR, with ROC curve areas of 0.7993 (95% CI = 0.6578–0.9408). Given the cut-off value of > 15.85 for the baseline PZP, the sensitivity was 83.33% (95% CI = 51.59%–97.91%) and the specificity was 76.32% (95% CI = 65.18%–85.32%), respectively.

The ROC curve areas for GELS and HEP2 were 0.7325(95% CI = 0.5993–0.8656) and 0.6952 (95% CI = 0.5784–0.8119), at the cutoff value of > 17.77 for GELS, > 18.06 for HEP2, the sensitivity and the specificity were 91.67(95% CI = 61.52%–99.79%) and 48.68 (95% CI = 37.04%–60.43%), 100 (95% CI = 73.54%–100%) and 47.37 (95% CI = 35.79%–59.16%), respectively.

The ROC curve areas for carcinoembryonic antigen (CEA) was 0.7337(95% CI = 0.5422–0.9252), at the cutoff value of < 2.935 for CEA, the sensitivity and the specificity were 75(95% CI = 34.91%–96.81%) and 76.09 (95% CI = 61.23%–87.41%), respectively (Fig. 1E).

We then used the Random Forest algorithm to build a machine learning-based statistical predictive model, which consisted of 10 protein candidates (PZP + CXCL7 + FINC + ZA2G + CO4A + S10A8 + KAIN + HEP2 + CO5 + GELS). The importance of each marker in the model was shown in Supplementary Table 6. Seventy percent of the data was used as the training set and the rest as the testing set. The AUC value is 0.972(95% CI = 0.969–0.976) on training set and 0.886(95% CI = 0.872–0.899) on testing data (Fig. 1F, G).

Therefore, our results indicated that the 10-protein panel was a useful predictive biomarker for differentiating pCR patients from non-pCR.

We also analyzed the correlation between the levels of candidate markers in post-nCRT and response. No statistically significant difference was found between the responding group and the non-responding group in post-nCRT sera. The levels of HEP2 and APOH in post-nCRT sera showed no significant difference between the responding group and the non-responding group (q = 0.515 and q = 0.258, respectively) (Supplementary Fig. 1E, F).

### Clinical pathological data and correlation with the levels of candidate biomarkers

Baseline patient characteristics, staging information and baseline level of PZP, GELS, HEP2, APOH, S10A8, PEDF and APOA1 for the 91 patients are shown in Table 2. The median age was 59 years with 63.7% male. Twelve (13.18%) patients achieved pathological complete response (ypT0N0), 8 (17.4%) patients experienced distant metastases and 3 patients (6.5%) deceased.

**Table 2:** Clinicopathological characteristics and recurrence, according to biomarkers level in pre-nCRT sera

Variable	APOA1 (*n* = 91)			APOH (*n* = 91)			S10A8 (*n* = 91)			HEP2 (*n* = 91)			GELS (*n* = 91)			PZP (*n* = 91)			PEDF(*n* = 91)		
	**Low *n* = 46**	**High *n* = 45**	**P**	**Low *n* = 46**	**High *n* = 45**	**P**	**Low *n* = 46**	**High *n* = 45**	**P**	**Low *n* = 46**	**High *n* = 45**	**P**	**Low *n* = 46**	**High *n* = 45**	**P**	**Low *n* = 46**	**High *n* = 45**	**P**	**Low *n* = 46**	**High *n* = 45**	**P**
**Age, years**																					
Median	**54**	**60**	**0.157**	**58**	**59.5**	**0.429**	**55**	**59.5**	**0.2286**	**59**	**58.5**	**0.5139**	**60**	**58**	**0.8310**	**55.46**	**59.26**	**0.0679**	**57.49**	**57.45**	**0.987**
Range	**9.9**	**8.19**		**9.91**	**9.53**		**9.827**	**9.509**		**9.83**	**9.62**		**9.792**	**9.708**		**9.98**	**9.169**		**9.818**	**9.687**	
**Gender**																					
Female	**14**	**19**	**0.280**	**16**	**17**	**1.00**	**16**	**17**	**0.8292**	**15**	**18**	**0.5173**	**15**	**17**	**0.6641**	**10**	**23**	**0.0046**	**18**	**15**	**0.6641**
Male	**32**	**26**		**28**	**30**		**30**	**28**		**31**	**27**		**31**	**28**		**36**	**22**		**28**	**30**	
**Pathological T stage**																					
ypT0-2	**13**	**29**	**0.0007**	**16**	**26**	**0.0361**	**17**	**25**	**0.0941**	**13**	**29**	**0.0007**	**12**	**30**	**0.0001**	**13**	**29**	**0.0007**	**15**	**27**	**0.0117**
ypT3-4	**33**	**16**		**30**	**19**		**29**	**20**		**33**	**16**		**34**	**15**		**33**	**16**		**31**	**18**	
**Pathological N stage**																					
ypN0	**23**	**37**	**0.0018**	**30**	**32**	**0.654**	**29**	**33**	**0.3696**	**27**	**35**	**0.0717**	**25**	**37**	**0.0065**	**31**	**31**	**1.0**	**27**	**35**	**0.0717**
ypN1-2	**23**	**8**		**16**	**13**		**17**	**12**		**19**	**10**		**21**	**8**		**15**	**14**		**19**	**10**	
**Pathological complete response**																					
Yes	**2**	**10**	**0.0141**	**6**	**6**	**1.0**	**4**	**8**	**0.2305**	**3**	**9**	**0.0695**	**2**	**10**	**0.0141**	**1**	**11**	**0.0017**	**4**	**8**	**0.2305**
No	**44**	**35**		**40**	**39**		**42**	**37**		**43**	**36**		**44**	**35**		**45**	**34**		**42**	**37**	
**Recurrence at any site**																					
Yes	**9**	**7**	**1.0**	**8**	**8**	**1.0**	**4**	**12**	**0.0295**	**10**	**6**	**0.41**	**10**	**6**	**0.41**	**8**	**8**	**1.0**	**9**	**7**	**0.784**
No	**37**	**38**		**38**	**37**		**42**	**33**		**36**	**39**		**36**	**39**		**38**	**37**		**37**	**38**	

High levels of PZP, GELS and APOA1 were associated with nCRT response (*p* = 0.0017, *p* = 0.014 and *p* = 0.014, respectively). High level of S10A8 was associated with metastases (*p* = 0.0295). The levels of APOA1, APOH, HEP2, GELS, PZP and PEDF were associated with neoadjuvant pathological tumor (ypT) stage. The levels of APOA1 and GELS were correlated with neoadjuvant pathological lymph node (ypN) stage (Table 2).

### Prognostic value of candidate biomarkers

**Fig. 2:**
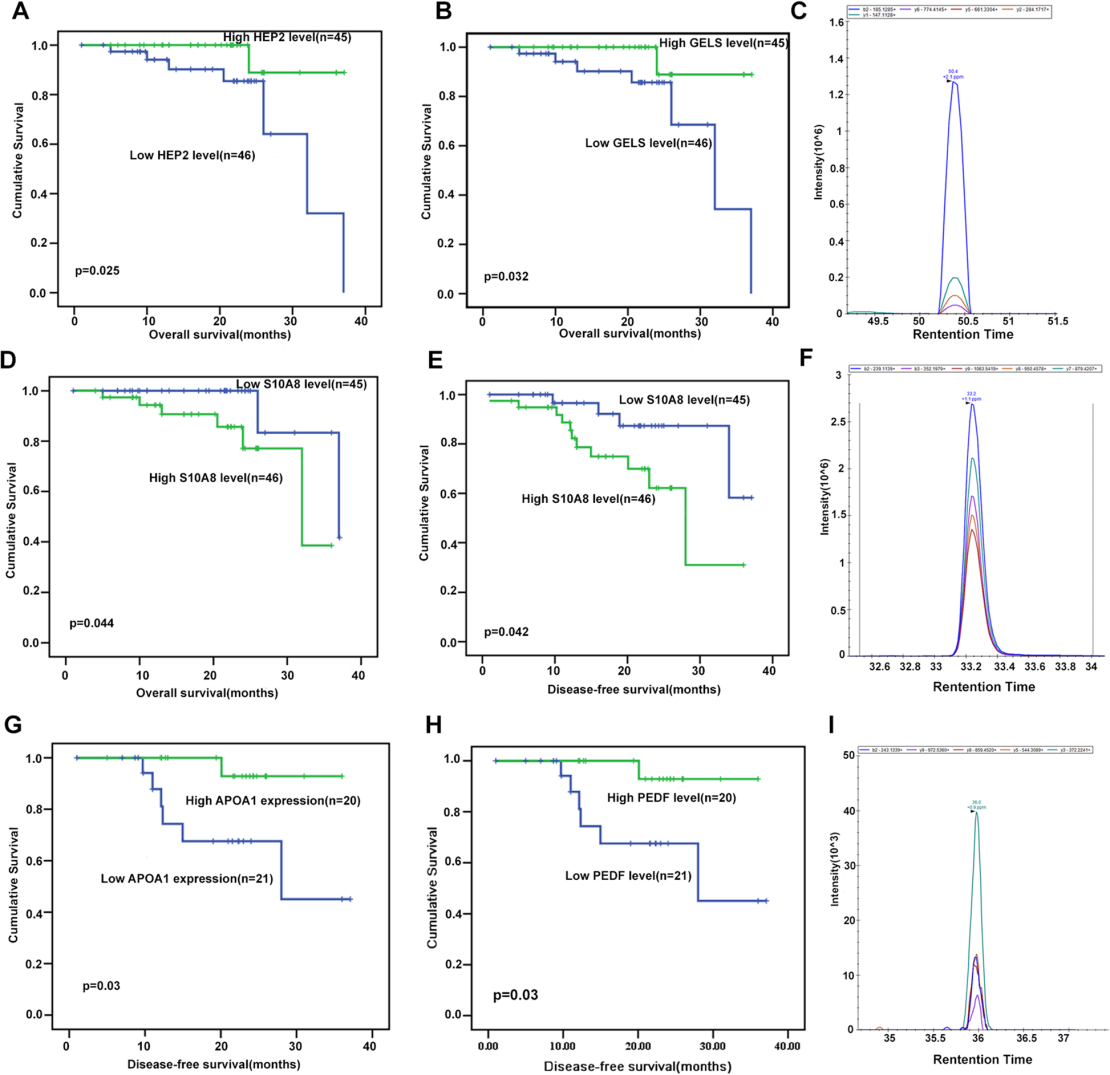
Kaplan–Meier analysis of the correlation between the candidate proteins’ level and prognosis in rectal cancer patients. **A**-**B** Kaplan–Meier analysis of the correlation among HEP2 and GELS baseline levels and OS in rectal cancer patients. **C**-**E** PRM traces for the quantification of S10A8 in baseline and its correlation with OS and DFS. **F**-**I** PRM traces for the quantification of APOA1 and PEDF in post-nCRT level and its correlation with DFS

The correlation among the candidate proteins’ baseline level and the clinical outcome was analyzed. Kaplan–Meier analyses for overall survival were performed using the median levels of proteins as the cutoff for the definition of the subgroups. Higher serum HEP2 and GELS or lower S10A8 were associated with better prognosis (*p* = 0.025, *p* = 0.032 and *p* = 0.044, respectively) (Fig. 2A-D). To determine whether there was prognostic association among the candidate proteins with DFS, we plotted Kaplan–Meier survival curves; the curves indicated a significant difference among DFS and the expression of baseline serum S10A8 (*p* = 0.042, the log–rank test) (Fig. 2E).

The correlation among the candidate proteins’ post-nCRT level and the clinical outcome was also examined. The results indicated that higher serum APOA1 and PEDF were associated with better DFS (*p* = 0.03) (Fig. 2F-I).

### Expression and prognostic value of candidate biomarkers in colorectal cancer tissues

**Fig. 3:**
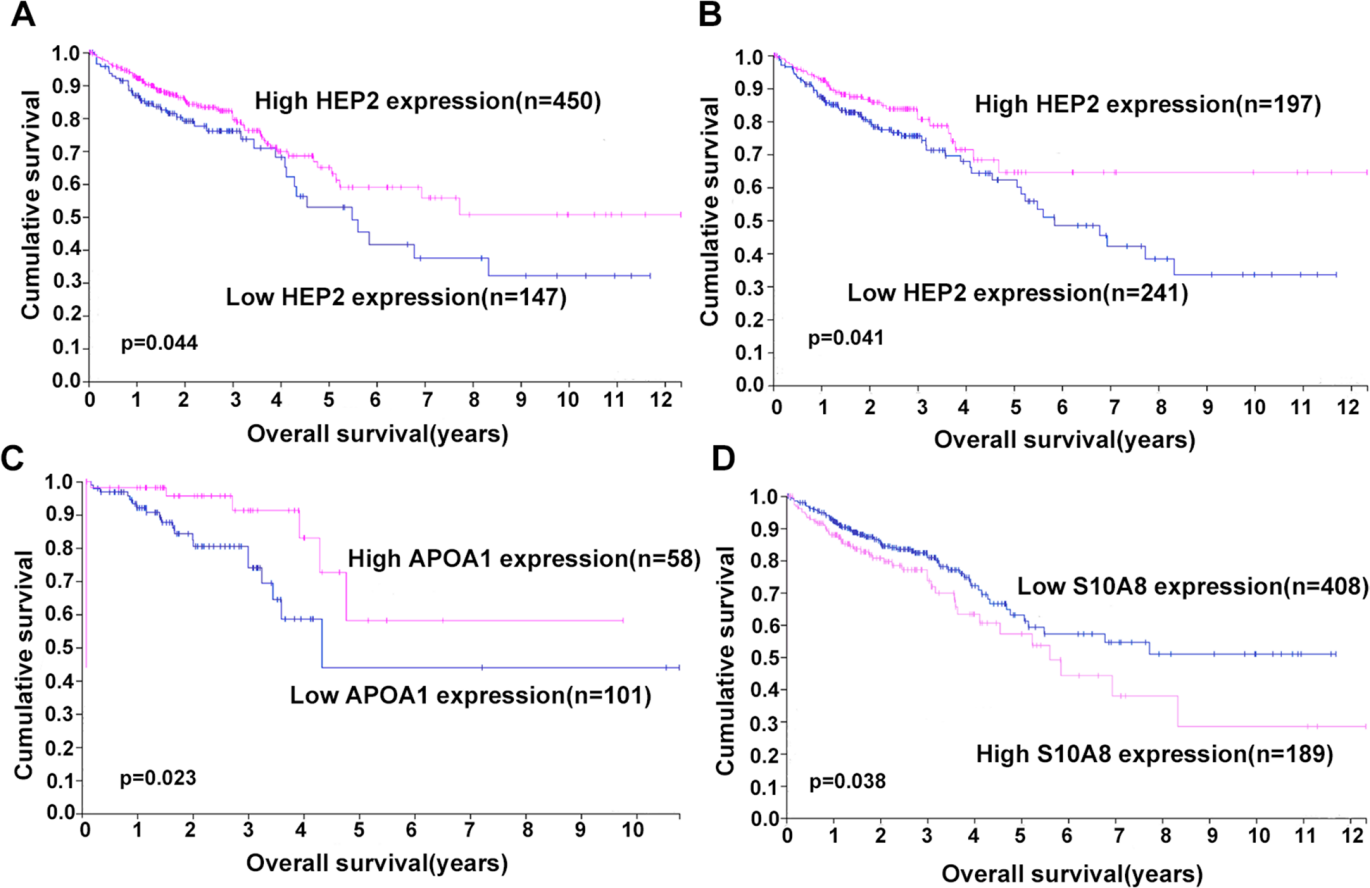
Kaplan–Meier estimates of OS of CRC patients according to several proteins level from TCGA CRC dataset. **A** The correlation between HEP2 and OS in CRC. **B** The correlation between HEP2 and OS in colon cancer. **C** The correlation between APOA1 and OS in rectal cancer. **D** The correlation between S10A8 and OS in CRC

In order to explore whether these candidate biomarkers play a potential role in CRC progress, we evaluated the prognostic significance of these markers in CRC tissues. We analyzed the correlation among their mRNA expressions and OS in the Cancer Genome Atlas (TCGA) CRC dataset. There are 597 subjects that have both survival and mRNA expression data, including 159 rectal cancers and 438 colon cancers. The survival analysis of CRC RNA-seq data from TCGA presented in the Human Protein Atlas (www.proteinatlas.org) showed that HEP2 expression was correlated with better prognosis of CRC (*p* = 0.044, Fig. 3A). HEP2 expression was shown to be significantly correlated with better prognosis of colon cancer (*p* = 0.041, Fig. 3B) but not rectal cancer. APOA1 expression was correlated with better prognosis of rectal cancer (*p* = 0.023, Fig. 3C) but not colon cancer. S10A8 expression negatively correlated with CRC prognosis (*p* = 0.038, Fig. 3D).

## Discussion

We analyzed pre- and post-nCRT sera samples of rectal cancer using an integrated work flow including iTRAQ-based comparative proteomics analysis and PRM-based targeted proteomic technique to identify and verify the proteins associated with nCRT therapeutic outcome. Our study indicated that the patients’ baseline immune condition could affect the response to nCRT. Immune activation-related proteins and pathways were enriched in the patients who were responding to the nCRT. The response LARC was characterized by classical antibody-mediated complement, FCGR activation, antigen activates B cell receptor, signaling by the B cell receptor (BCR) and innate immune system.

Tumor regression and downstaging after neoadjuvant treatment are known prognostic factors for survival in rectal cancer, which were used as endpoints to evaluate neoadjuvant treatment effect [20]. Patients achieving pathological complete response have lower rates of local and distant disease recurrence. Variable responses to nCRT among tumors might be due to different tumor biology and or tumor microenvironment. Tumor regression after preoperative CRT is a multifaceted phenomenon. Many researchers proposed that it might be associated with smaller, less aggressive disease, and may also correspond to the molecular tumor profile regulating treatment response [21].

Recent studies have shown that the immune-mediated components contribute to chemotherapy and radiotherapy efficacy. High infiltration of CD8^+^ cells in tumor biopsies predominated in the subgroup of responders (complete or partial response) to nCRT, whereas the majority of nonresponders showed a low infiltration of CD8 + cells [22]. Depletion of T cells or neutralization of interferon-gamma reversed radiation-induced equilibrium leading to tumor regrowth [23]. It was observed that radio-responsive tumors exhibited greater intratumoral immune activity than non-responsive tumors in a murine colon tumor model. The responder tumors have increased numbers of immune cells as well as elevated levels of intratumoral IFNγ and CD8 + T cells. In essence, non-responder tumors presented very similar to control unirradiated tumors [24]. nCRT might be considered as an immune adjuvant acting through both the innate and adaptive immune responses. Radiation which kills tumor cells through various aspects may act synergistically with the immune system, each making the other more efficient [25].

Based on the high-throughput PRM validation, a panel of proteins was observed and the results of the Random Forest algorithm highlight the superiority of a 10-protein panel for the prediction of response to nCRT in rectal cancer. The most contributing factor in the prediction model is PZP. PZP is up-regulated in a number of different inflammatory conditions and may contribute to immune regulation by non-covalently sequestering a series of other ligands including TNF-alpha, IL-2 and IL-6 [26, 27]. In our study, PZP was shown to be correlated with nCRT response and ypT stage. PZP might be able to promote tumor regression through interaction with these ligands.

We further investigated the prognostic value of the panel of proteins in rectal cancer. The levels of S10A8, HEP2 and GELS in baseline sera were correlated with prognosis of rectal cancer. There was no significant correlation between PZP level and prognosis. There are multiple clinical and histopathologic factors that are relevant in affecting prognosis following nCRT. TRG alone is not definitive. The ypT and ypN category remain the most important prognostic factors [28]. We also noted that the levels of HEP2 and GELS correlated with ypT, GELS correlated with ypN, while the level of S10A8 correlated with distant metastasis. It was reported that the protease-induced uncovering of cryptic epitopes in HEP2 could transform the molecule into a host defense factor [29]. In our study, the expression of HEP2 in CRC tissues was correlated with DFS and OS, indicating that its potential inhibitory role in CRC recurrence.

GELS expression is downregulated in various cancers such as colorectal cancer and gastric cancers [30, 31] and GELS overexpression could inhibit human colorectal cancer cell invasion and migration [31], indicating its tumor suppressing role. GELS is involved in regulating changes in actin dynamics that affect cell survival signaling pathways and intestinal inflammation [32]. Plasma GELS interacts with various immune system cell types. It is involved in the regulation and adhesion of neutrophils and is able to enhance the function of peripheral T-cells, leading to a decrease of inflammatory cytokines like IL-1β and IL-6 in the brain[33]. GELS might promote tumor regression and inhibit metastasis in rectal cancer after nCRT through its direct role on tumor cells or indirect effect on immune cells.

In our study, sera S10A8 level was found to be associated with tumor recurrence and negatively correlated with OS and DFS in rectal cancer after nCRT. We have noticed that S10A8 expression in CRC tissues negatively correlated with OS. It was reported that S10A8 was expressed to a greater extent in colorectal, prostate and breast cancers [34–36]. In colorectal cancers, high expression of S10A8 correlated with Dukes stage, liver and lymph node metastasis and poor prognosis [37]. S10A8 was reported to be mainly expressed by immune cells within tumors, and their expression can stimulate the recruitment of myeloid [38] and myeloid-derived suppressor cells, resulting in pre-metastatic niche formation, tumor growth and metastasis [39]. In the metastatic liver microenvironment, monocytes/macrophages induced the expression of S10A8 in CRC cells, which promoted tumor cell migration and invasion [40]. A positive correlation was found between S10A8 expression and PD-L1 expression in human colorectal cancer specimens. S10A8 induced PD-L1 expression in monocytes/macrophages and attenuated the antitumor ability of CTLs both in vitro and in a CT26 tumor–bearing mouse tumorigenesis model [41]. Our results further indicate that tumors with higher original immune activity tend to further evoke anti-tumor immune response induced by chemoradiotherapy, resulting in a better response to CRT.

In this work, we demonstrated that APOA1 and PEDF in post-nCRT sera were correlated with DFS of rectal cancer. It is reported that high serum APOA1 level has been associated with a decreased risk of several cancers including colorectal adenomas and CRC [42]. Low serum APOA1 levels were associated with advanced T stage and TNM-stage in CRC. Serum APOA1 level showed strong negative correlation with CRP and interleukin IL-8 levels and blood neutrophil count [43]. Our previous work showed that patients with high PEDF expression after nRT had better DFS in rectal cancer [44]. This study further implicated that PEDF might be a prognostic marker.

Two studies similar to ours have also focused on identifying proteins that predict the response of patients with rectal cancer to neoadjuvant chemoradiotherapy. Dayde D. et al. performed pretreatment plasma proteome of a mouse model of rectal cancer treated with concurrent chemoradiation [45]. Plasma VEGFR3 was identified as a potential biomarker to predict the nCRT response. They also found that the predictive power of combining VEGFR3, EGFR and COX2 was improved compared with the performance of each biomarker, with an AUC of 0.869 (sensitivity of 43% at 95% specificity). In their research, the proteomic study using tumor bearing mouse blood samples instead of human blood samples provided another research perspective on the one hand, and also brought the difference with human blood samples on the other hand. In addition, they were verified in a smaller human blood sample.

In the study of Chauvin A. et al. [46]*,* they analyzed the proteome of FFPE biopsies using an in-gel separation followed by HPLC–MS/MS and showed several proteins that overexpressed in total responders or non-responders. After H&E staining, the pathologist selected the area of interest and make a punch in the paraffin block to obtain FFPE biopsy. Different sample types and sampling methods make the research results vary among different studies.

Limitations of the study are retrospective design, the limited number of cases and lack of independent cohort validation. An independent cohort validation would further consolidate the predictive and prognostic value. Future prospective studies with a large cohort are required to further confirm the clinical implications of these biomarkers in rectal cancer studies. Another limitation is that we evaluated the expression and prognostic value of candidate biomarkers in CRC tissues using CRC tissues RNA-seq data from TCGA data. It is more suitable to analyze these markers in rectal cancer tissues with nCRT, so as to analyze their relationship with chemoradiotherapy sensitivity and prognosis. We intend to do it in our future work.

## Conclusions

In our study, a 10-protein panel was identified for the prediction of response to nCRT and several potential biomarkers were identified for prognosis of rectal cancer based on iTRAQ-based comparative proteomics screening and PRM-based targeted proteomic validation. The elevated level of S10A8 was correlated with metastasis and poorer OS. The decreased levels of HEP2 and GELS were correlated with inferior OS. The levels of APOA1 and PEDF in post nCRT were positively correlated with DFS.

Our findings suggest that baseline immune activity affect the response to nCRT. The identified proteins and protein panel in this study may serve as potential biomarkers for predicting response to nCRT or prognosis of rectal cancer following nCRT. We believe that our findings will enable to provide forecasting clues to whether rectal cancer patients will benefit from nCRT and help to stratify patients who are at the greatest risk of distant metastasis and tailor their adjuvant therapy.

## Supplementary Information

**Additional file 1:** **Supplementary Table 1.** Clinical features of serum samples for iTRAQ experiment. **Supplementary Table 2.** Clinical features of tested serum samples. **Supplementary Table 3.** List of proteins identified and quantified across all the individual sera samples. **Supplementary Table 4.** List of significant differential proteins between different groups identified by iTRAQ. **Supplementary Table 5.** The pepetides used for quantification: characteristics and performance. **Supplementary Table 6.** The importance of each feature in predition model.

**Additional file 2:** **Supplementary Figure 1.** Sera levels of several proteins in response and resistant to nCRT groups of rectal cancer. (A-B) Scatter plots of baseline sera GELS and HEP2 concentrations obtained from pCR and non-pCR patients using the PRM. (C-D) Scatter plots of baseline sera GELS and HEP2 concentrations obtained from the responding and the non-responding patients using the PRM. (E-F) Scatter plots of post-nCRT sera HEP2 and APOH concentrations obtained from the responding and the non-respondingpatients using the PRM.

## Data Availability

The mass spectrometry proteomics data have been deposited to the ProteomeXchange Consortium (http://proteomecentral.proteomexchange.org) via the iProX partner repository with the dataset identifier PXD032117.

## Abbreviations

nCRT: Neoadjuvant chemoradiation treatment
iTRAQ: Isobaric tags for relative and absolute quantitation
PRM: Parallel-reaction monitoring
pCR: Pathological complete response
DFS: Disease-free survival
MS: Mass spectrometry
MRM: Multiple-reaction monitoring
TRG: Tumor regression grade
ROC: Receiver operating characteristic curve
OS: Overall survival
mRNA: Messenger RNA
CRC: Colorectal cancer
TCGA: The Cancer Genome Atlas
RNA-seq: RNA sequencing
LARC: Locally advanced rectal cancer
IFN: Interferin
TNF: Tumor necrosis factor
IL: Interleukin
CRP: C-reactive protein
CT: Computed tomography
TME: Total mesorectal excision
IMRT: Intensity-modulated radiation therapy
CTV: Clinical target volume
CEA: Carcinoembryonic antigen
EDRN: Early Detection Research Networks
FASP: Filter-aided sample preparation
TCEP: Tris-(2-carboxyethyl) phosphine
IAA: Iodoacetamide
FA: Formic acid
MS: Mass spectrometry
LC: Liquid chromatography
HPLC: High performance liquid chromatography
DDA: Data-dependent acquisition
XIC: Extracted ion chromatogram
AUC: Area under the curve
