# Supplementary material for: Predictive value of proteomic markers for advanced rectal cancer with neoadjuvant chemoradiotherapy
Source: BMC Cancer. 2022 Aug 9;22:868. doi: 10.1186/s12885-022-09960-z (PMC9361520; doi:10.1186/s12885-022-09960-z)
Supplement: Supplementary file 2 — Additional file 2: Supplementary Figure 1. Sera levels of several proteins in response and resistant to nCRT groups of rectal cancer. (A-B) Scatter plots of baseline sera GELS and HEP2 concentrations obtained from pCR and non-pCR patients using the PRM. (C-D) Scatter plots of baseline sera GELS and HEP2 concentrations obtained from the responding and the non-responding patients using the PRM. (E-F) Scatter plots of post-nCRT sera HEP2 and APOH concentrations obtained from the responding and the non-respondingpatients using the PRM. [file 12885_2022_9960_MOESM2_ESM.pdf]

Supplementary Figure 1.

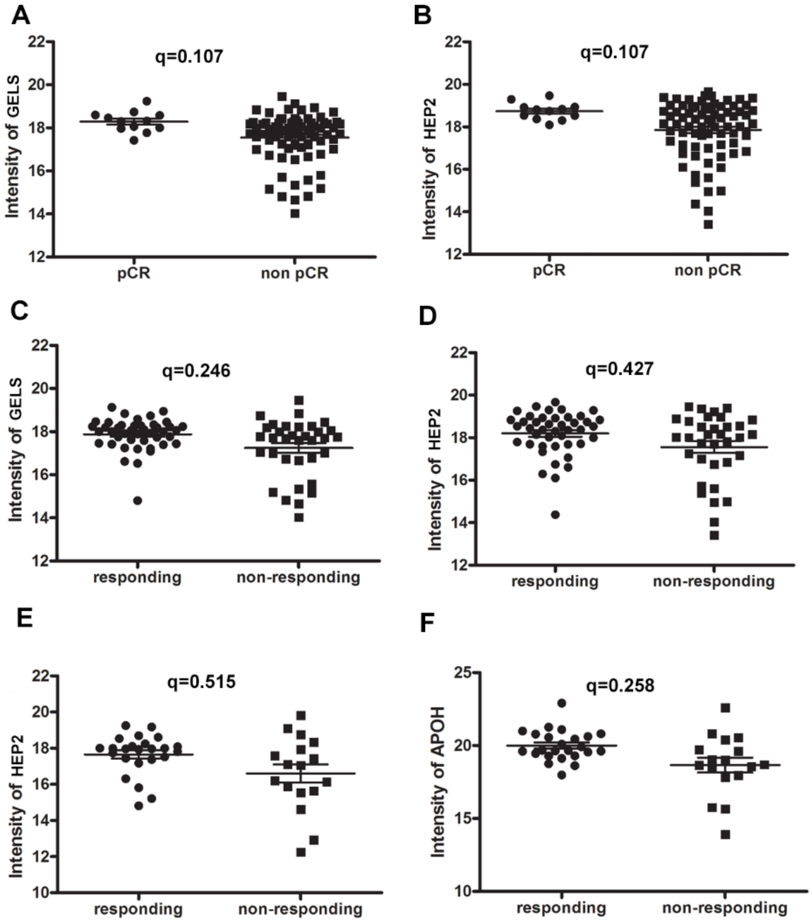

Supplementary Figure 1. Sera levels of several proteins in response and resistant to nCRT groups of rectal cancer. (A-B) Scatter plots of baseline sera GELS and HEP2 concentrations obtained from pCR and non-pCR patients using the PRM. (C-D) Scatter plots of baseline sera GELS and HEP2 concentrations obtained from the responding and the non-responding patients using the PRM. (E-F) Scatter plots of post-nCRT sera HEP2 and APOH concentrations obtained from the responding and the non-responding patients using the PRM.
